# An in-depth analysis of young adults with osteonecrosis secondary to developmental dysplasia of the hip who underwent total hip arthroplasty

**DOI:** 10.1186/s12891-024-07517-8

**Published:** 2024-06-04

**Authors:** Sandeep Krishan Nayar, Avi Marks, Aresh Hashemi-Nejad, Andreas Roposch

**Affiliations:** 1 Great Ormond Street Hospital, Great Ormond Street, London, WC1N 3JH UK; 2 Royal National Orthopaedic Hospital, Brockley Hill, Stanmore, HA7 4LP UK; 3 UCL Institute of Child Health, 30 Guildford Street, London, WC1N 3EH UK

**Keywords:** DDH, Osteonecrosis, Total hip arthroplasty

## Abstract

**Background:**

Patients with osteonecrosis of the femoral head secondary to DDH frequently require total hip arthroplasty (THA), but it is not well understood which factors necessitate this requirement. We determined the incidence of THA in patients who have osteonecrosis secondary to DDH and factors associated with need for THA.

**Methods:**

We included patients who received closed or open reductions between 1995 and 2005 with subsequent development of osteonecrosis. We determined osteonecrosis according to Bucholz and Ogden; osteoarthritis severity (Kellgren-Lawrence), subluxation (Shenton’s line); neck-shaft angle; and acetabular dysplasia (centre-edge and Sharp angles). We also recorded the number of operations of the hip in childhood and reviewed case notes of patients who received THA to describe clinical findings prior to THA. We assessed the association between radiographic variables and the need for THA using univariate logistic regression.

**Results:**

Of 140 patients (169 hips), 22 patients received 24 THA (14%) at a mean age of 21.3 ± 3.7 years. Associated with the need for THA were grade III osteonecrosis (OR 4.25; 95% CI 1.70-10.77; *p* = 0.0019), grade IV osteoarthritis (21.8; 7.55–68.11; *p* < 0.0001) and subluxation (8.22; 2.91–29.53; *p* = 0.0003). All patients who required THA reported at least 2 of: severe pain including at night, stiffness, and reduced mobility. Acetabular dysplasia and number of previous operations were not associated with the need for THA.

**Conclusions:**

We identified a 14% incidence of THA by age 34 years in patients with osteonecrosis secondary to DDH. Grade III osteonecrosis (global involvement femoral head and neck) was strongly associated with THA, emphasising the importance to avoid osteonecrosis when treating DDH.

**Supplementary Information:**

The online version contains supplementary material available at 10.1186/s12891-024-07517-8.

## Background

Osteonecrosis of the femoral head is a well-recognised complication in the treatment of developmental dysplasia of the hip (DDH), with a reported incidence of up to 73% after closed or open reduction [1–5]. The sequalae following osteonecrosis includes proximal femoral growth disturbance, femoral head collapse and inhibition of acetabular remodelling, all of which can predispose to early onset osteoarthritis.

In prior research we showed that young adults with osteonecrosis secondary to DDH demonstrated minimal overall physical disability and a normal quality of life; however their hip function was reduced in the presence of osteonecrosis grades III and IV according to Bucholz and Ogden [6]. This previous study excluded patients that had received total hip arthroplasty (THA). To date, there is no literature concerning factors necessitating THA in patients with osteonecrosis secondary to open or closed reductions in DDH.

Whilst modern THA implants in young adults have shown promising functional outcomes and revision rates [7, 8], it is not without its risks and there is high chance of requiring revision in one’s lifetime. It is therefore important to understand the incidence of THA following this complication and the risk factors that necessitate it. This will help us to better inform our patients as well as identify those who might be at higher risk, which may influence surveillance and treatment options to improve hip biomechanics and delay need for THA [9].

The aims of this study were to describe [1] the incidence of THA in patients with osteonecrosis secondary to DDH [2], associations between radiographic parameters and the need for THA, and [3] the characteristics of patients who underwent THA.

## Methods

The local Research Ethics Committee approved this study (REC 14/LO/1267). Written informed consent was obtained from all patients over the age of 16 years and written informed consent was obtained from parent/guardians of those under the age of 16 years.

We included patients with a diagnosis of DDH who had received a closed reduction or open reduction with or without osteotomy between 1995 and 2005 and who subsequently developed osteonecrosis. All patients were treated in two tertiary centres and were identified from our previous study [6] with no change in the study population.

**Fig. 1:**
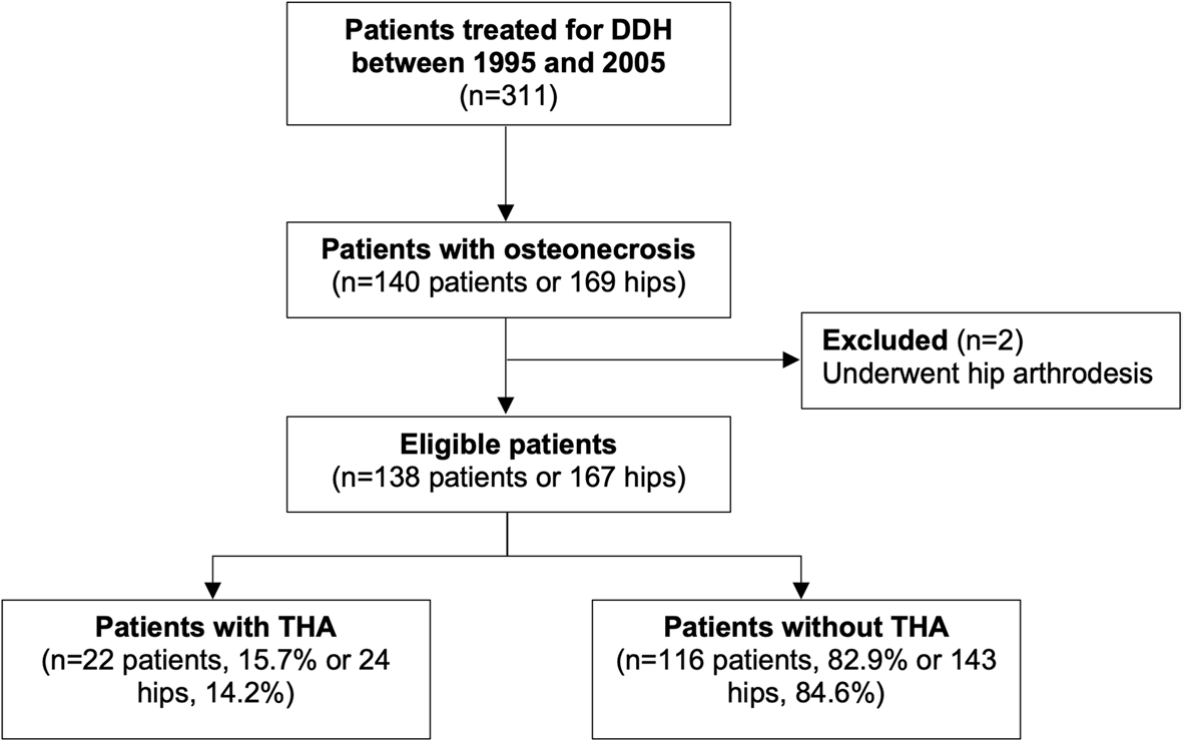
Flow diagram showing patient eligibility and participation. THA, total hip arthroplasty

Of 140 patients included, 29 had bilateral osteonecrosis, encompassing a total of 169 hips. Of these, 24 hips (14%) proceeded to arthroplasty, whereas two patients received bilateral THA (Fig. 1). The mean age at the time of THA was 21.3 years (range, 16–29 years) (Table 4 in Appendix 1). Another two patients (1%) underwent hip arthrodesis at ages 9 and 12 years. These patients were not representative of the study group and therefore excluded from further analysis. We compared patient-characteristics including sex, laterality, number of prior operations and age at study between patients with and without THA.

**Fig. 2:**
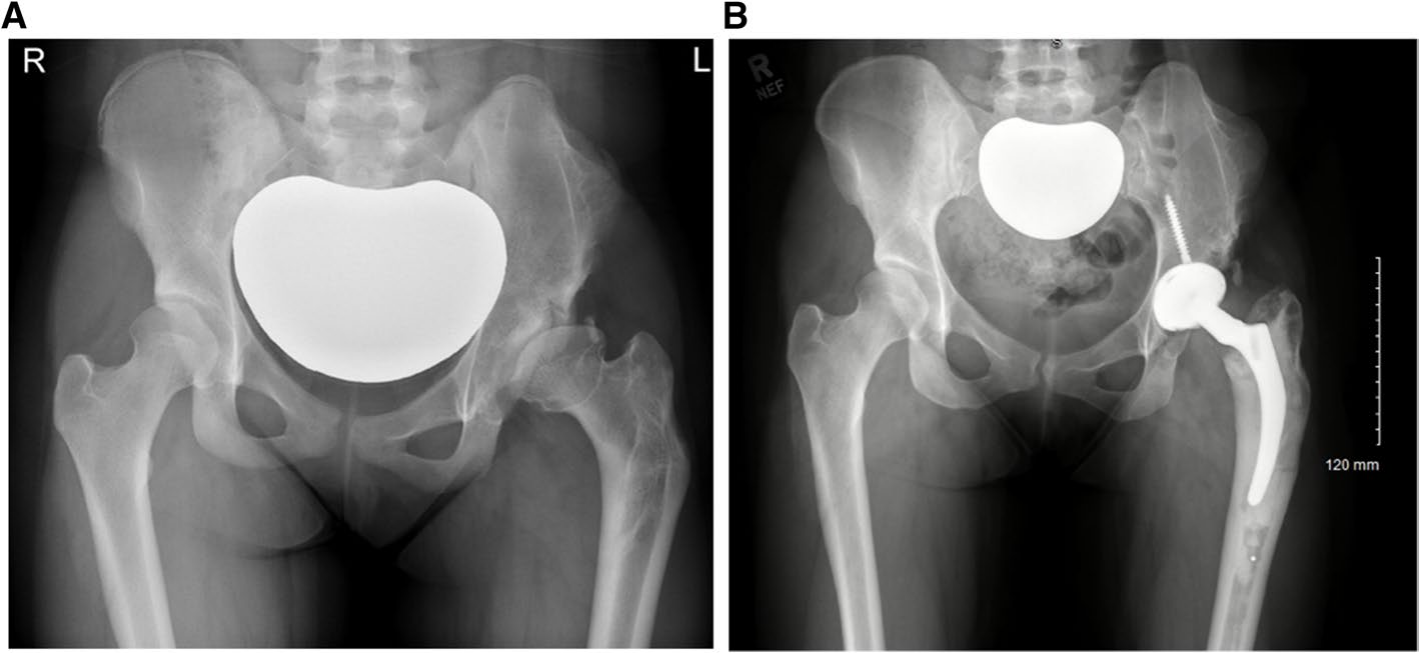
**A** A pelvic radiograph obtained 15 years after open reduction, Salter innominate osteotomy and femoral varus de-rotation osteotomy. It shows grade III osteonecrosis of the left hip in a 17-year-old girl. There is total femoral head involvement, marked acetabular dysplasia and subluxation of the hip. She had a positive Trendelenburg gait and 1 cm difference in leg lengths. **B** Pelvic radiograph of the same patient 5 years post THA, performed at age 18 years

**Fig. 3:**
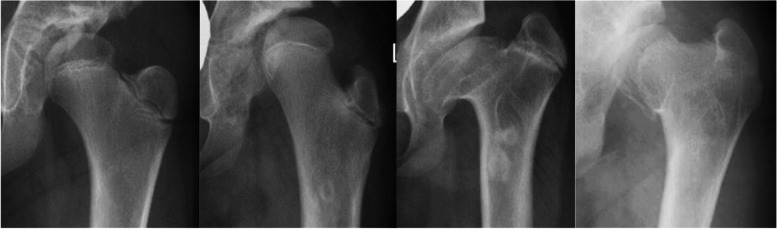
Radiographs representing Grade I, Grade II, Grade III, and Grade IV (from left to right) of the Bucholz-Ogden classification for osteonecrosis secondary to DDH

All participants had a standing AP pelvic radiograph at the time of our previous study assessment in 2017 [6]. In cases where THA had already been undertaken, their latest AP pelvic radiograph prior to hip replacement was used. In one case radiographs prior to THA were not available, therefore radiographic analysis was only performed for 23 hips in the THA group. All radiographs were carried out using a standardised protocol on a digital imaging system (GE Medical Systems Ltd., Buckinghamshire, UK) (Fig. 2). Grade of osteonecrosis was assessed according to Bucholz-Ogden [10]. Grade I changes are limited to the femoral head with hypoplasia of the femoral head but normal ossification of the metaphysis. In Grade II, the lateral metaphysis is injured and the femoral head will grow into valgus. For Grade III, the entire metaphysis is involved resulting in shortening of the femoral neck with trochanteric overgrowth. An injury or defect along the medial metaphysis is present in Grade IV causing varus of the proximal femur (Fig. 3). Osteoarthritis was graded according to Kellgren-Lawrence [11]. Acetabular dysplasia was quantified by measuring the centre-edge angle of Wiberg [12] and the acetabular angle of Sharp [13]. All radiographs were assessed by two independent reviewers as outlined previously [6]. Interrater reliability was deemed excellent for the centre-edge and acetabular angles (intra-class correlation coefficient = 0.86), and moderate for the Kellgren-Lawrence (κ = 0.62) and Bucholz-Ogden (κ = 0.64) grading. Consensus was obtained with the senior author (AR) in the case of any disagreements [6]. All patients that underwent THA had their clinical assessment and surgery under the same surgeon (AHN).

Clinical information for the patients that received THA was collected by reviewing the electronic patient records, specifically looking at factors that may influence need for THA. This included the nature of their previous operations of the same hip, co-morbidities [14], medications [15], smoking status [16], drug or alcohol abuse [17, 18], clinical symptoms prior to THA such as pain and walking ability, and examination findings prior to THA [19, 20]. In two cases the exact nature of their previous operations could not be identified.

We compared groups using chi square or Fisher’s exact test and Student’s t test as appropriate. We used univariable logistic regression to examine associations between radiographic characteristics and need for THA. Because ‘subluxation’ is a known risk factor for early hip failure, we tested the effect of osteonecrosis adjusted for subluxation using Firth’s penalised likelihood estimates [21]. We estimated the cumulative occurrence of THA over time using Kaplan-Meier survival analysis. We repeated the analysis by randomly excluding of one side in bilateral cases to assess non-independence in these patients, which did not result in any significant change to the reported results [22, 23]. In sensitivity analyses we accounted for potentially missed cases of THA (e.g. those performed in other hospitals, thus unknown to us): we repeated the analyses and randomly selected subjects) into the THA group at 17%, 20%, 22% and 25%; this did not change the estimates of effect. All data were analysed using GraphPad® Prism 9 (GraphPad Software, California, USA).

## Results

**Fig. 4:**
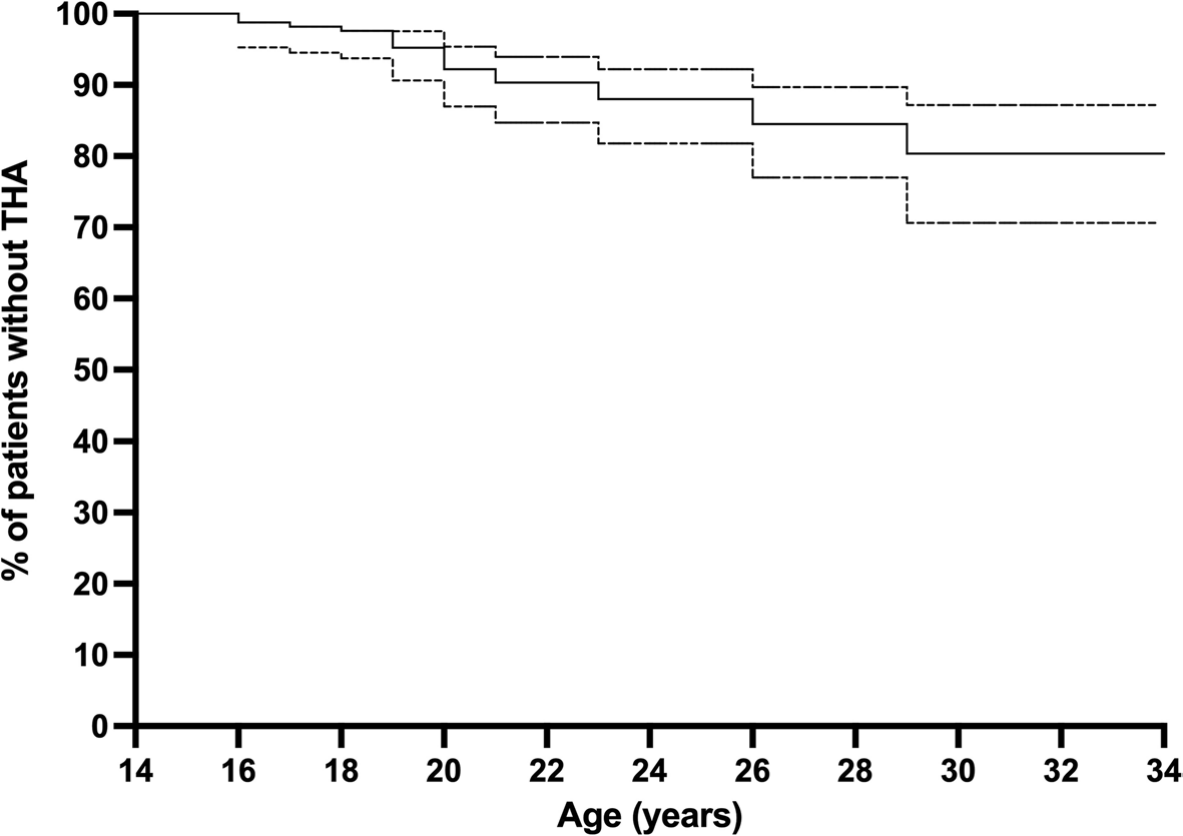
Graph showing the cumulative occurrence of THA (solid line) and 95% confidence interval (dotted line)

By age 34 years 24 THA (14%) were observed, two patients (9%) underwent bilateral THA (Fig. 4). In one case these were performed one month apart, and in the other they were performed 28 months apart. One patient (4%) had bilateral osteonecrosis where only a single side warranted THA.

Patients with THA were older at the time of study assessment by 3 ± 1 years (*p* = 0.0003); but they were similar in terms of sex (*p* = 0.308), laterality (*p* = 0.635) and number of prior operations (*p* = 0.227) (Table 1).

**Table 1:** Group differences based on univariate analysis

Variable	Patients with THA (*n* = 22)	Patients without THA (*n* = 116)	*P* value
Mean age at time of study (range)	29 years (21 – 33)	26 years (21 – 34)	0.0003
Sex, n (%)			0.308
Male	1 (5%)	16 (14%)	
Female	21 (95%)	100 (86%)	
DDH laterality, n (%)			0.635
Unilateral	15 (71%)	71 (61%)	
Bilateral	7 (29%)	45 (39%)	
Mean number of operations prior to THA, n (range)	2 (1 – 9)	2 (1 – 5)	0.227

Factors associated with THA included osteonecrosis of grade III (OR 4.25, 95% CI 1.70–10.77; *p* = 0.0019); osteoarthritis of grade IV (OR 21.78, 95% CI 7.55–68.11; *p* < 0.0001); and subluxation (OR 8.22, 95% CI 2.91–29.53, *p* = 0.0003) (Table 2). A lower neck-shaft angle was weakly associated with THA (OR 0.96, 95% CI 0.93–0.99; *p* = 0.0262), which was lost on sensitivity analysis beyond an assumption of any additional patients undergoing THA. Markers of acetabular dysplasia were not associated with THA. The effect of grade III osteonecrosis remained (*p* = 0.0026) when the analysis was adjusted for subluxation of the hip.

**Table 2:** Radiographic results based on univariate analysis

Variable	Patients with THA (*n* = 23 hips)	Patients without THA (*n* = 143 hips)	OR (95% CI)	*P* value
**Osteonecrosis grade, *n* (%)**				
I	4 (17%)	14 (10%)	1.93 (0.51–6.05)	0.289
II	4 (17%)	84 (60%)	0.15 (0.041–0.41)	0.0008
III	12 (52%)	29 (20%)	4.25 (1.70–10.77)	0.0019
IV	3 (13%)	15 (10%)	1.27 (0.28–4.29)	0.724
**Degree of osteoarthritis, n (%)**				
0	0 (0%)	31 (22%)	Not converged	0.0085
I	0 (0%)	51 (36%)	Not converged	0.0001
II	6 (26%)	37 (26%)	0.60 (0.17–1.72)	0.376
III	4 (17%)	15 (10%)	1.78 (0.47–5.54)	0.347
IV	13 (57%)	8 (6%)	21.8 (7.55–68.11)	< 0.0001
Lateral centre-edge angle, degrees (mean ± SD)	13.3^o^ ± 13.5	18.9^o^ ± 12.9	0.97 (0.93–1.00)	0.0589
Sharp’s acetabular angle, degrees (mean ± SD)	46.5^o^ ± 5.9	44.8^o^ ± 6.3	1.05 (0.97–1.12)	0.215
Shenton’s line broken, n (%)	19 (83%)	52 (36%)	8.22 (2.91–29.53)	0.0003
Neck shaft angle, degrees (mean ± SD)	128^o^ ± 14.2	134^o^ ± 12	0.96 (0.93–0.99)	0.0262

Of the hips requiring THA, 11 hips (46%) were reduced closed and 10 (42%) were reduced open. Two hips (8%) received proximal femoral osteotomy only, four hips (17%) received pelvic osteotomy only and eight hips (33%) underwent both proximal femoral and pelvic osteotomy (Table 3). There was no identified association between those that received closed versus open reduction and grade of osteonecrosis or osteoarthritis.

Thirteen patients (59%) had no co-morbidities, five patients (23%) had obesity (BMI > 30), one patient had hypermobility, one had IgA nephropathy following Henoch-Schönlein Purpura, one had asthma and one had bilateral talipes equinovarus. One patient was a smoker (20 cigarettes per day). There was no mention of alcohol or illicit drug use in any of these cases. Average BMI was 25 (range 17 to 41) (Table 3).

Nineteen patients (86%) had reported severe pain prior to THA, including night pain in 16 patients (72%). In the remaining three patients, two were predominantly troubled by stiffness and one experienced pain in conjunction with a 5 cm leg length discrepancy. Reduced mobility, with an inability to walk beyond one mile or 10 min, was reported in 17 cases (77%). A positive Trendelenburg test indicating poor abductor function was reported in six cases (Table 3).

**Table 3:** Characteristics of 22 patients at the time of receiving THA

Variable	Frequency
Co-morbidities	
None, n (%)	13 (59.1%)
Obesity (BMI > 30), n (%)	5 (22.7%)
Asthma, n (%)	1 (4.5%)
Hypermobility, n (%)	1 (4.5%)
IgA nephropathy, n (%)	1 (4.5%)
Talipes Equinovarus, n (%)	1 (4.5%)
Smoker, n (%)	1 (4.5%)
Alcohol use, n (%)	0
Illicit drug use, n (%)	0
Body mass index (mean ± SD)	25 ± 6
Index surgery	
Closed reduction, n (%)	11 (45.8%)
Open reduction, n (%)	10 (41.7%)
Unknown, n (%)	3 (12.5%)
Osteotomies prior to THA	
Proximal femur, n (%)	2 (8%)
Pelvic, n (%)	4 (17%)
Combined, n (%)	8 (33%)
Unknown, n (%)	2 (8%)
Clinical symptoms	
Severe pain, n (%)	19 (86.4%)
Night pain, n (%)	16 (72.7%)
Mobility < 1 mile or 10 min, n (%)	17 (77.3%)
Fixed flexion deformity, degrees (mean ± SD) (*n* = 6)	15^o^ ± 9
Positive Trendelenburg, n (%) (*n* = 11)	6 (54.5%)
Leg length discrepancy, cm (mean ± SD) (*n* = 14)	1.5 cm ± 1.2
Range of motion	
Flexion, degrees (mean ± SD) (*n* = 20)	83^o^ ± 20
Abduction (*n* = 14)	19^o^ ± 14
Adduction (*n* = 13)	10^o^ ± 9
Internal Rotation (*n* = 19)	10^o^ ± 14
External Rotation (*n* = 20)	18^o^ ± 16

## Discussion

Osteonecrosis, or physeal arrest, is a serious complication in the treatment of DDH and occurs in up to 73% of cases [1–5]. Whilst its pathogenesis is unknown, risks factors include age at index surgery [24], perioperative injury to the proximal femoral blood supply [25], and an eccentric position of the femoral head in plaster [26]. Our previous study showed overall high scores in patient-reported outcomes at a mean age of 21 years [6]. However, in 18 patients their function was so poor that they had received THA. Since then, a further four patients in this study population have also deteriorated to the point of needing a THA. We wanted to conduct an in-depth analysis of these 24 patients in order to discern common features resulting in THA.

14% of our patients required a THA by age 34 years, some of which were performed as early as 16 years of age. Naturally, the dominating features necessitating THA included severe osteoarthritis in 74% of cases, subluxation of the hip in 83% of cases and grade III osteonecrosis in 52%.

Grade III osteonecrosis according to Bucholz-Ogden is characterised by complete physeal arrest with femoral neck shortening (coxa breva), femoral head flattening (coxa plana) and relative overgrowth of the greater trochanter. It is thus regarded the most severe form of osteonecrosis, not only in terms of morphology but also in terms of functional outcomes [2, 27]. Furthermore, slower acetabular remodelling has previously been observed in dysplastic hips with grade III osteonecrosis [28].

There was no association between grade IV osteonecrosis and the need for THA. This may be confounded by the relatively smaller number of patients with this grade of osteonecrosis in this study.

In contrast, grade II osteonecrosis, which is characterised by lateral physeal arrest and coxa valga, had a protective effect against need for THA. One explanation for this is that such hips would have been more likely to receive varus osteotomies of the proximal femur leading to improved femoral head coverage and joint congruency. This, in turn, would optimise the biomechanics with reduced stress loading and instability [28].

A broken Shenton’s line has been demonstrated to be an accurate radiographic predictor of femoral head subluxation [29]. This has been linked to increased acetabular lateral edge loading and subsequent development of osteoarthritis [30], hence resulting in increased need for THA. From our dataset, 78% of all patients with grade III or IV osteoarthritis displayed a subluxated hip.

A lower neck shaft angle was weakly associated with need for THA. However, this association was lost during sensitivity analysis. Coxa vara in the context of osteonecrosis is frequently associated with additional morphological changes including coxa plana, trochanteric overgrowth and leg length discrepancy, all of which can contribute to development of osteoarthritis, rather than lower NSA being an independent risk factor in itself [31].

Notably, the association between acetabular dysplasia and the need for THA was of borderline statistical significance in this study. Roposch et al. [32] established that in hips with DDH, osteonecrosis reduced acetabular remodelling to a degree that was linked with an increased risk for osteoarthritis.

These factors combined result in development of osteoarthritis. Severe osteoarthritis subsequently results in increasing pain and stiffness, with resultant reduction in mobility, function, and overall quality of life. Validity studies have demonstrated a clear relationship between radiographic Kellgren-Lawrence score, clinical symptoms of hip osteoarthritis including pain and reduced range of motion, and need for THA [33, 34]. Clinically, patients in our cohort required THA due to severe and debilitating hip pain including at night, stiffness, and significant reduction in mobility (to less than 1 mile or 10 min).

Osteonecrosis can result in overgrowth of the greater trochanter with resultant shortening of the abductor lever arm and hence abductor muscle weakness. Whilst some patients in this group had a positive Trendelenburg test to indicate poor abductor function, this was not a clear theme for all patients that underwent THA. Hence, poor abductor function does not appear to be an independent factor associated with need for THA.

There are limitations to this study. Notably, comparative data was only available for radiographic information and number of previous operations, and could not be obtained for clinical parameters due to the retrospective nature of this study. Radiographic markers were limited to a single snapshot in time. The Bucholz-Ogden classification of osteonecrosis secondary to DDH has been reported to have variable interrater reliability, particularly when distinguishing between grades I and II, therefore results should be interpreted with caution [35]. Furthermore, the study design only allows for association rather than true causal relationship. Bias from bilateral cases and potential missed cases was accounted for with no significant change in the reported results.

Whilst this study provides some interesting insights into the factors associated with increased need for THA, further prospective data collection over time is warranted to better explore these associations.

## Conclusions

This study identified a 14% incidence of THA by age 34 years in patients with osteonecrosis secondary to DDH following previous closed or open reduction. Associated factors include Bucholz-Ogden grade III osteonecrosis, Kellgren-Lawrence grade IV osteoarthritis and subluxation of the hip. Hips at risk of THA should be reviewed more closely and if symptomatic, discussed with a young adult hip unit with respect to whether further hip preservation surgery should be undertaken so as not to compromise the results of a THA. Furthermore, these findings emphasise the importance to avoid osteonecrosis when treating DDH.

### Supplementary Information

Supplementary Material 1.

## Data Availability

The datasets used and/or analysed during the current study are available from the corresponding author on reasonable request.

## Appendix 1

**Table 4:** Individual cases that received THA

ID	Age at THA	Sex	Bilateral DDH	Bucholz-Ogden	Kellgren-Lawrence	Sharp’s acetabular angle	Lateral CE angle	Shenton’s line	No. ops prior to THA	BMI	Co-morbidities	Clinical symptoms prior to THA	ROM prior to THA	LLD (cm)	Trendelenburg	Smoker
1	19	F	No	3	4	54	23	Broken	1	26	CTEV	Severe pain including at night + stiffness	F: 80^o^ Ab: - Ad: - ER: - IR: -	1	-	No
2	23	F	No	3	4	45	23	Intact	1	25	Nil	Severe pain including at night + reduced mobility	F: 80^o^ Ab: - Ad:10^o^ ER: 20^o^ IR: 10^o^	1	-	No
3	21	F	No	3	3	43	12	Intact	1	31	Nil	Severe pain including at night + reduced mobility	F: 80^o^ Ab: 20^o^ Ad: 0^o^ ER: 10^o^ IR: 10^o^	2	-	No
4	19	F	No	3	4	51	0	Broken	1	27	Nil	Severe pain including at night + reduced mobility (walking distance < 1 mile)	F: 90^o^ Ab: 10^o^ Ad: - ER: 20^o^ IR: 45^o^	1	-	No
5	19	F	Yes	3	4	42	27	Broken	2	25	Nil	Severe pain including at night + reduced mobility	F: 80^o^ Ab: 20^o^ Ad: 10^o^ ER: 30^o^ IR: 0^o^	-	-	No
6	19	F	No	3	4	47	16	Broken	2	20	Nil	Pain, stiffness + reduced mobility (walking distance < 1 mile)	F: 80^o^ Ab: 20^o^ Ad: 10^o^ ER: 15^o^ IR: 0^o^	2.5 cm	-ve	No
7	29	F	Yes	3	4	51	7	Broken	2	34	Nil	Severe pain + reduced mobility	F: 70^o^ Ab: - Ad: - ER: 0^o^ IR: 0^o^	-	-ve	No
8	17	F	No	3	2	49	-10	Intact	1	20	Nil	Pain at night + marked LLD	F: 70^o^ Ab: 0^o^ Ad: 100^o^ ER: 0^o^ IR: 0^o^	5 cm	+ve	No
9	29	M	Yes	3	3	37	28	Broken	3	18	Nil	Severe pain including at night + reduced mobility	F: - Ab: - Ad: - ER: - IR: -	1	-ve	No
10	16	F	No	3	3	48	22	Broken	9	24	Asthma	Severe pain including at night + reduced mobility	F: 95^o^ Ab: 25^o^ Ad: 5^o^ ER: 45^o^ IR: 10^o^	2	-	No
11	26	F	No	3	4	50	-10	Broken	1	20	Nil	Severe pain + stiffness	F: 110^o^ Ab: 25^o^ Ad: 20^o^ ER: 60^o^ IR: 40^o^	-	+ve	No
12	26	F	Yes	2	2	51	0	Broken	3	28	Nil	Severe pain including at night + reduced mobility (walking time < 5 min)	F: 60^o^ Ab:20^o^ Ad: 10^o^ ER: 20^o^ IR: 0^o^	-	-	No
13	20	F	No	2	2	53	0	Broken	1	24	Nil	Severe pain including at night + reduced mobility	F: 120^o^ Ab: 50^o^ Ad: 30^o^ ER: 10^o^ IR: 30^o^	0.5	-	No
14	20	F	No	4	2	48	21	Broken	4	18	Nil	Severe pain including at night + reduced mobility (walking time < 10 min)	F: - Ab: - Ad: - ER: - IR: -	-	-	No
15	18	F	Yes	4	4	57	14	Broken	8	20	Nil	Severe pain including at night + reduced mobility (walking time < 10 min)	F: 90^o^ Ab: 20^o^ Ad: - ER: 5^o^ IR: 20^o^	-	+ve	No
16	20	F	No	1	4	34	16	Broken	1	24	Nil	Severe pain + reduced mobility	F: 100^o^ Ab: - Ad: - ER: 20^o^ IR: 10^o^	0.5	-ve	No
17	23	F	No	1	3	44	12	Broken	5	27	Nil	Severe pain including at night + reduced mobility (walking time < 5 min)	F: 80^o^ Ab: - Ad:10^o^ ER: 30^o^ IR: -	-	-	No
18	21	F	No	1	4	52	3	Broken	1	20	IgA nephropathy	Severe pain + reduced mobility	F: 110^o^ Ab: 30^o^ Ad: 20^o^ ER: 30^o^ IR: 10^o^	2	-	No
19	21	F	No	1	4	43	33	Broken	2	40	Nil	Severe pain including at night, stiffness + reduced mobility	F: - Ab: - Ad: - ER: 0^o^ IR: 0^o^	-	+ve	No
20	16	F	No	-	-	-	-	-	1	17	Nil	Severe pain including at night + reduced mobility (walking distance < 1 mile)	F: - Ab: - Ad: - ER: - IR: -	-	-	No
21	26	F	Yes	2	2	35	29	Intact	1	19	Hypermobility	Severe pain including at night + reduced mobility (walking time < 10 min)	F: 90^o^ Ab: 30^o^ Ad: 30^o^ ER: 30^o^ IR: 5^o^	-	-ve	20/d
	23			2	2	44	31	Broken				Severe pain including at night + clicking	F: 90^o^ Ab: - Ad: - ER: 20^o^ IR: 5^o^	0.5	-	
22	20	F	Yes	4	4	44	-10	Broken	1	34	Nil	Severe pain including at night + reduced mobility	F: 45^o^ Ab: 0^o^ Ad: - ER: 0^o^ IR: 0^o^	1	+ve	No
	20			3	4	48	18	Broken				Severe pain including at night + reduced mobility	F: 45^o^ Ab: 0^o^ Ad: 0 ^o^ ER: 0^o^ IR: 0^o^	1	+ve	

## References

[1] Brougham DI, Broughton NS, Cole WG, Menelaus MB (1988). The predictability of acetabular development after closed reduction for congenital dislocation of the hip. J Bone Joint Surg Br.

[2] Roposch A, Liu LQ, Offiah AC, Wedge JH (2011). Functional outcomes in children with osteonecrosis secondary to treatment of developmental dysplasia of the hip. J Bone Joint Surg Am.

[3] Brougham DI, Broughton NS, Cole WG, Menelaus MB (1990). Avascular necrosis following closed reduction of congenital dislocation of the hip. Review of influencing factors and long-term follow-up. J Bone Joint Surg Br.

[4] Kalamchi A, MacEwen GD (1980). Avascular necrosis following treatment of congenital dislocation of the hip. J Bone Joint Surg Am.

[5] Cooperman DR, Wallensten R, Stulberg SD (1980). Post-reduction avascular necrosis in congenital dislocation of the hip. J Bone Joint Surg Am.

[6] Marks A, Cortina-Borja M, Maor D, Hashemi-Nejad A, Roposch A (2021). Patient-reported outcomes in young adults with osteonecrosis secondary to developmental dysplasia of the hip - a longitudinal and cross-sectional evaluation. BMC Musculoskelet Disord.

[7] Walker RP, Gee M, Wong F, Shah Z, George M, Bankes MJ (2016). Functional outcomes of total hip arthroplasty in patients aged 30 years or less: a systematic review and meta-analysis. Hip Int.

[8] Buddhdev PK, Vanhegan IS, Khan T, Hashemi-Nejad A (2020). Early to medium-term outcomes of uncemented ceramic-bearing total hip arthroplasty in teenagers for paediatric hip conditions. Bone Joint J.

[9] Bayliss LE, Culliford D, Monk AP, Glyn-Jones S, Prieto-Alhambra D, Judge A (2017). The effect of patient age at intervention on risk of implant revision after total replacement of the hip or knee: a population-based cohort study. Lancet.

[10] Bucholz RW (1978). The Hip Proceedings of the Sixth Open Scientific Meeting of the Hip Society. Patterns of ischemic necrosis of the proximal femur in nonoperatively treated congenital hip disease.

[11] Kellgren JH, Lawrence JS (1957). Radiological assessment of osteo-arthrosis. Ann Rheum Dis.

[12] Fredensborg N (1976). The CE angle of normal hips. Acta Orthop Scand.

[13] Sharp IK (1961). Acetabular dysplasia. J Bone Joint Surg Br Vol.

[14] Calders P, Van Ginckel A (2018). Presence of comorbidities and prognosis of clinical symptoms in knee and/or hip osteoarthritis: a systematic review and meta-analysis. Semin Arthritis Rheum.

[15] Cui B, Chen Y, Tian Y, Liu H, Huang Y, Yin G (2022). Effects of medications on incidence and risk of knee and hip joint replacement in patients with osteoarthritis: a systematic review and meta-analysis. Adv Rheumatol.

[16] Felson DT, Zhang Y (2015). Smoking and osteoarthritis: a review of the evidence and its implications. Osteoarthritis Cartilage.

[17] To K, Mak C, Zhang C, Zhou Y, Filbay S, Khan W (2021). The association between alcohol consumption and osteoarthritis: a meta-analysis and meta-regression of observational studies. Rheumatol Int.

[18] Ramczykowski T, Kruppa C, Schildhauer TA, Dudda M (2018). Total hip arthroplasty following illicit drug abuse. Arch Orthop Trauma Surg.

[19] Dreinhöfer KE, Dieppe P, Stürmer T, Gröber-Grätz D, Flören M, Günther KP (2006). Indications for total hip replacement: comparison of assessments of orthopaedic surgeons and referring physicians. Ann Rheum Dis.

[20] Crawford RW, Murray DW (1997). Total hip replacement: indications for surgery and risk factors for failure. Ann Rheum Dis.

[21] Firth D (1993). Bias reduction of maximum likelihood estimates. Biometrika.

[22] Park MS, Kim SJ, Chung CY, Choi IH, Lee SH, Lee KM (2010). Statistical consideration for bilateral cases in orthopaedic research. J Bone Joint Surg Am.

[23] LeBrun DG, Tran T, Wypij D, Kocher MS (2019). Statistical analysis of dependent observations in the orthopaedic sports literature. Orthop J Sports Med.

[24] Okano K, Yamada K, Takahashi K, Enomoto H, Osaki M, Shindo H (2009). Long-term outcome of Ludloff’s medial approach for open reduction of developmental dislocation of the hip in relation to the age at operation. Int Orthop.

[25] Ucar DH, Isiklar ZU, Stanitski CL, Kandemir U, Tumer Y (2004). Open reduction through a medial approach in developmental dislocation of the hip: a follow-up study to skeletal maturity. J Pediatr Orthop.

[26] Koizumi W, Moriya H, Tsuchiya K, Takeuchi T, Kamegaya M, Akita T (1996). Ludloff’s medial approach for open reduction of congenital dislocation of the hip. A 20-year follow-up. J Bone Joint Surg Br.

[27] Pollet V, Bonsel J, Ganzeboom B, Sakkers R, Waarsing E (2021). Morphological variants to predict outcome of avascular necrosis in developmental dysplasia of the hip. Bone Joint J.

[28] Millis MB, Kim YJ (2002). Rationale of osteotomy and related procedures for hip preservation: a review. Clin Orthop Relat Res.

[29] Rhee PC, Woodcock JA, Clohisy JC, Millis M, Sucato DJ, Beaulé PE (2011). The Shenton line in the diagnosis of acetabular dysplasia in the skeletally mature patient. J Bone Joint Surg Am.

[30] Song K, Pascual-Garrido C, Clohisy JC, Harris MD (2021). Acetabular edge loading during gait is elevated by the anatomical deformities of hip dysplasia. Front Sports Act Living.

[31] Zhang B, Sun J, Du Y, Shen J, Li T, Zhou Y (2021). Treatment of osteoarthritis secondary to severe coxa vara with modular total hip arthroplasty. Ther Clin Risk Manag.

[32] Roposch A, Ridout D, Protopapa E, Nicolaou N, Gelfer Y (2013). Osteonecrosis complicating developmental dysplasia of the hip compromises subsequent acetabular remodeling. Clin Orthop Relat Res.

[33] Reijman M, Hazes JM, Pols H, Bernsen R, Koes B, Bierma-Zeinstra S (2004). Validity and reliability of three definitions of hip osteoarthritis: cross sectional and longitudinal approach. Ann Rheum Dis.

[34] Birrell F, Croft P, Cooper C, Hosie G, Macfarlane G, Silman A (2001). Predicting radiographic hip osteoarthritis from range of movement. Rheumatology.

[35] Roposch A, Wedge JH, Riedl G (2012). Reliability of Bucholz and Ogden classification for osteonecrosis secondary to developmental dysplasia of the hip. Clin Orthop Relat Res.

