# Supplementary material for: An in-depth analysis of young adults with osteonecrosis secondary to developmental dysplasia of the hip who underwent total hip arthroplasty
Source: BMC Musculoskelet Disord. 2024 Jun 4;25:436. doi: 10.1186/s12891-024-07517-8 (PMC11149231; doi:10.1186/s12891-024-07517-8)
Supplement: Supplementary file 1 — Supplementary Material 1. [file 12891_2024_7517_MOESM1_ESM.docx]

**Appendix 1:** Individual cases that received THA

| **ID** | **Age at THA** | **Sex** | **Bilateral DDH** | **Bucholz-Ogden** | **Kellgren-Lawrence** | **Sharp’s acetabular angle** | **Lateral CE angle** | **Shenton’s line** | **No. ops prior to THA** | **BMI** | **Co-morbidities** | **Clinical symptoms prior to THA** | **ROM prior to THA** | **LLD (cm)** | **Trendelenburg** | **Smoker** |
| --- | --- | --- | --- | --- | --- | --- | --- | --- | --- | --- | --- | --- | --- | --- | --- | --- |
| 1 | 19 | F | No | 3 | 4 | 54 | 23 | Broken | 1 | 26 | CTEV | Severe pain including at night + stiffness | F: 80^o^  Ab: -  Ad: -  ER: -  IR: - | 1 | - | No |
| 2 | 23 | F | No | 3 | 4 | 45 | 23 | Intact | 1 | 25 | Nil | Severe pain including at night + reduced mobility | F: 80^o^  Ab: -  Ad:10^o^  ER: 20^o^  IR: 10^o^ | 1 | - | No |
| 3 | 21 | F | No | 3 | 3 | 43 | 12 | Intact | 1 | 31 | Nil | Severe pain including at night + reduced mobility | F: 80^o^  Ab: 20^o^  Ad: 0^o^  ER: 10^o^  IR: 10^o^ | 2 | - | No |
| 4 | 19 | F | No | 3 | 4 | 51 | 0 | Broken | 1 | 27 | Nil | Severe pain including at night + reduced mobility (walking distance <1 mile) | F: 90^o^  Ab: 10^o^  Ad: -  ER: 20^o^  IR: 45^o^ | 1 | - | No |
| 5 | 19 | F | Yes | 3 | 4 | 42 | 27 | Broken | 2 | 25 | Nil | Severe pain including at night + reduced mobility | F: 80^o^  Ab: 20^o^  Ad: 10^o^  ER: 30^o^  IR: 0^o^ | - | - | No |
| 6 | 19 | F | No | 3 | 4 | 47 | 16 | Broken | 2 | 20 | Nil | Pain, stiffness + reduced mobility (walking distance <1 mile) | F: 80^o^  Ab: 20^o^  Ad: 10^o^  ER: 15^o^  IR: 0^o^ | 2.5cm | -ve | No |
| 7 | 29 | F | Yes | 3 | 4 | 51 | 7 | Broken | 2 | 34 | Nil | Severe pain + reduced mobility | F: 70^o^  Ab: -  Ad: -  ER: 0^o^  IR: 0^o^ | - | -ve | No |
| 8 | 17 | F | No | 3 | 2 | 49 | -10 | Intact | 1 | 20 | Nil | Pain at night + marked LLD | F: 70^o^  Ab: 0^o^  Ad: 100^o^  ER: 0^o^  IR: 0^o^ | 5cm | +ve | No |
| 9 | 29 | M | Yes | 3 | 3 | 37 | 28 | Broken | 3 | 18 | Nil | Severe pain including at night + reduced mobility | F: -  Ab: -  Ad: -  ER: -  IR: - | 1 | -ve | No |
| 10 | 16 | F | No | 3 | 3 | 48 | 22 | Broken | 9 | 24 | Asthma | Severe pain including at night + reduced mobility | F: 95^o^  Ab: 25^o^  Ad: 5^o^  ER: 45^o^  IR: 10^o^ | 2 | - | No |
| 11 | 26 | F | No | 3 | 4 | 50 | -10 | Broken | 1 | 20 | Nil | Severe pain + stiffness | F: 110^o^  Ab: 25^o^  Ad: 20^o^  ER: 60^o^  IR: 40^o^ | - | +ve | No |
| 12 | 26 | F | Yes | 2 | 2 | 51 | 0 | Broken | 3 | 28 | Nil | Severe pain including at night + reduced mobility (walking time <5 mins) | F: 60^o^  Ab:20^o^  Ad: 10^o^  ER: 20^o^  IR: 0^o^ | - | - | No |
| 13 | 20 | F | No | 2 | 2 | 53 | 0 | Broken | 1 | 24 | Nil | Severe pain including at night + reduced mobility | F: 120^o^  Ab: 50^o^  Ad: 30^o^  ER: 10^o^  IR: 30^o^ | 0.5 | - | No |
| 14 | 20 | F | No | 4 | 2 | 48 | 21 | Broken | 4 | 18 | Nil | Severe pain including at night + reduced mobility (walking time <10 mins) | F: -  Ab: -  Ad: -  ER: -  IR: - | - | - | No |
| 15 | 18 | F | Yes | 4 | 4 | 57 | 14 | Broken | 8 | 20 | Nil | Severe pain including at night + reduced mobility (walking time <10 mins) | F: 90^o^  Ab: 20^o^  Ad: -  ER: 5^o^  IR: 20^o^ | - | +ve | No |
| 16 | 20 | F | No | 1 | 4 | 34 | 16 | Broken | 1 | 24 | Nil | Severe pain + reduced mobility | F: 100^o^  Ab: -  Ad: -  ER: 20^o^  IR: 10^o^ | 0.5 | -ve | No |
| 17 | 23 | F | No | 1 | 3 | 44 | 12 | Broken | 5 | 27 | Nil | Severe pain including at night + reduced mobility (walking time <5 mins) | F: 80^o^  Ab: -  Ad:10^o^  ER: 30^o^  IR: - | - | - | No |
| 18 | 21 | F | No | 1 | 4 | 52 | 3 | Broken | 1 | 20 | IgA nephropathy | Severe pain + reduced mobility | F: 110^o^  Ab: 30^o^  Ad: 20^o^  ER: 30^o^  IR: 10^o^ | 2 | - | No |
| 19 | 21 | F | No | 1 | 4 | 43 | 33 | Broken | 2 | 40 | Nil | Severe pain including at night, stiffness + reduced mobility | F: -  Ab: -  Ad: -  ER: 0^o^  IR: 0^o^ | - | +ve | No |
| 20 | 16 | F | No | - | - | - | - | - | 1 | 17 | Nil | Severe pain including at night + reduced mobility (walking distance <1 mile) | F: -  Ab: -  Ad: -  ER: -  IR: - | - | - | No |
| 21 | 26 | F | Yes | 2 | 2 | 35 | 29 | Intact | 1 | 19 | Hypermobility | Severe pain including at night + reduced mobility (walking time <10 mins) | F: 90^o^  Ab: 30^o^  Ad: 30^o^  ER: 30^o^  IR: 5^o^ | - | -ve | 20/d |
|  | 23 |  |  | 2 | 2 | 44 | 31 | Broken |  |  |  | Severe pain including at night + clicking | F: 90^o^  Ab: -  Ad: -  ER: 20^o^  IR: 5^o^ | 0.5 | - |  |
| 22 | 20 | F | Yes | 4 | 4 | 44 | -10 | Broken | 1 | 34 | Nil | Severe pain including at night + reduced mobility | F: 45^o^  Ab: 0^o^  Ad: -  ER: 0^o^  IR: 0^o^ | 1 | +ve | No |
|  | 20 |  |  | 3 | 4 | 48 | 18 | Broken |  |  |  | Severe pain including at night + reduced mobility | F: 45^o^  Ab: 0^o^  Ad: 0 ^o^  ER: 0^o^  IR: 0^o^ | 1 | +ve |  |
